# A three-gene signature as potential predictive biomarker for irinotecan sensitivity in gastric cancer

**DOI:** 10.1186/1479-5876-11-73

**Published:** 2013-03-22

**Authors:** Jie Shen, Jia Wei, Hao Wang, Guofeng Yue, Lixia Yu, Yang Yang, Li Xie, Zhengyun Zou, Xiaoping Qian, Yitao Ding, Wenxian Guan, Baorui Liu

**Affiliations:** 1 The Comprehensive Cancer Centre of Drum Tower Hospital, Medical School of Nanjing University, Clinical Cancer Institute of Nanjing University, 321 Zhongshan Rd, Nanjing, 210008, China; 2 Department of General Surgery, Drum Tower Hospital, Medical School of Nanjing University, Clinical Cancer Institute of Nanjing University, 321 Zhongshan Rd, Nanjing, 210008, China; 3 Nanjing University of Traditional Chinese Medicine, 138 Xianlin Rd, Nanjing, 210029, China

**Keywords:** Personalized chemotherapy, Irinotecan, Gastric cancer, HDRA, Immunodeficient mice

## Abstract

**Objective:**

Personalized chemotherapy based on molecular biomarkers can maximize anticancer efficiency. We aim to investigate predictive biomarkers capable of predicting response to irinotecan-based treatment in gastric cancer.

**Methods:**

We examined gene expression of APTX, BRCA1, ERCC1, ISG15, Topo1 and methylation of SULF2 in formalin-fixed paraffin-embedded gastric cancer tissues from 175 patients and evaluated the association between gene expression levels or methylation status and *in vitro* sensitivity to irinotecan. We used multiple linear regression analysis to develop a gene-expression model to predict irinotecan sensitivity in gastric cancer and validated this model *in vitro* and *vivo*.

**Results:**

Gene expression levels of APTX, BRCA1 and ERCC1 were significantly lower in irinotecan-sensitive gastric cancer samples than those irinotecan-resistant samples (*P* < 0.001 for all genes), while ISG15 (*P* = 0.047) and Topo1 (*P* = 0.002) were significantly higher. Based on those genes, a three-gene signature were established, which was calculated as follows: Index =0.488 - 0.020× expression level of APTX + 0.015× expression level of Topo1 - 0.011 × expression level of BRCA1. The three-gene signature was significantly associated with irinotecan sensitivity (rho = 0.71, *P* < 0.001). The sensitivity and specificity for the prediction of irinotecan sensitivity based on the three-gene signature reached 73% and 86%, respectively. In another independent testing set, the irinotecan inhibition rates in gastric samples with sensitive-signature were much higher than those with resistant-signature (65% vs. 22%, *P* < 0.001). Irinotecan therapy with 20 mg/kg per week to immunodeficient mice carrying xenografts with sensitive-signature dramatically arrested the growth of tumors (*P* < 0.001), but had no effect on mice carrying xenografts with resistant-signature.

**Conclusions:**

The three-gene signature established herein is a potential predictive biomarker for irinotecan sensitivity in gastric cancer.

## Introduction

Gastric cancer remains one of the leading causes of cancer death worldwide [1,2]. There is no “gold standard” chemotherapy for advanced gastric cancer up to now. In recent years, new generation chemotherapy agents, such as docetaxel, oxaliplatin, irinotecan, capecitabine and S-1 have been studied in phase III studies [3]. However, the median survival remained below one year [2], and the response rate was only approximately 30%-50% [4]. Irinotecan (CPT-11) is a semisynthetic derivative of camptothecin (CPT) which interferes with DNA replication and cell division through its potent interaction with the enzyme topoisomerase I (Topo1). Both irinotecan and CPT belong to Topo1 inhibitors. Irinotecan is mainly used in colorectal cancer and also frequently used in the treatment of gastric cancer, showing response rates varying from 14% to 23% as single agent and approximately 50% in combination [5].

A number of molecular biomarkers capable of predicting the probability of response to chemotherapeutic agents have been investigated over the last decades [6]. Our previous studies have identified breast cancer susceptibility gene 1 (BRCA1) as potential predictive biomarker for cisplatin and docetaxel sensitivity [7,8], thymidylate synthase (TS) for 5-FU and raltitrexed [9,10], and excision repair cross-complementing 1 (ERCC1) for platinum [11]. However, there has been limited progress in the identification of biomarkers capable of predicting response to irinotecan-based treatment in gastric cancer. Topo1 regulates DNA supercoiling during replication through the way of causing single-strand breaks and religation [12]. Irinotecan and its active metabolite SN-38 induce DNA damage by stabilizing a transient covalent complex between DNA and Topo1, which then results in DNA strand breaks, replication arrest, and apoptosis [13]. High tumor levels of Topo1 protein have recently been reported to identify a subgroup of metastatic colorectal cancer patients with good response to irinotecan [14]. Repair of irinotecan-associated and Topo1-mediated DNA damage requires removal of the stalled Topo1 and resolution of the associated DNA break. During this process, a variety of repair proteins, including aprataxin (APTX), BRCA1 and ERCC1, are involved, some of which may have clinical potential as predictive biomarkers [15].

Besides of the candidate biomarkers mentioned above, recent studies suggested that the methylation of the heparan sulfate 6-O-endosulfatase (SULF2) promoter was associated with sensitivity to Topo1 inhibitors in Non-Small-Cell Lung Cancer (NSCLC) [16]. INF-inducible regulator of ubiquitination (ISG15) could block the ubiquitin/26S proteasomal pathway leading to accumulation of CPT-induced DNA damage which resulted in an increased apoptosis [17]. SULF2 methylation (SULF2M) and high ISG15 expressing NSCLC cell lines showed 134-fold sensitivity to CPT than SULF2 unmethylation (SULF2U) and low ISG15 expressing cell lines [16].

Based on the above evidences, we hypothesized that APTX, BRCA1, ERCC1, ISG15, SULF2, and Topo1 or their combination might play important roles in predicting irinotecan sensitivity in gastric cancer. In the current study, we investigated each gene as predictive biomarker by itself, and then established algorithm combining those genes together to more accurately predict irinotecan sensitivity. We also validated the model in another independent set of gastric cancer samples and two cohorts of immunodeficient mice models. The aim of this study was to identify a clinically useful classification signature that could predict the irinotecan sensitivity in gastric cancer.

## Materials and methods

### Patient samples

All specimens and relevant clinical data were obtained from the department of oncology and general surgery, Drum Tower Hospital Affiliated to Medical School of Nanjing University during the period from August 2010 to June 2012. The specimens include 175 freshly-removed gastric tumors, which were randomly classified as either training set (n = 100) or testing set (n = 75) by using computer-generated random numbers. Each tumor tissue was divided into two parts once it removed in the surgery: (1) one part was kept in 4°C Hanks’ balanced salt solution with 1% penicillin/streptomycin and detected chemosensitivity *in vitro* by histoculture drug response assay (HDRA); (2) the rest part was left in formalin and made into formalin-fixed paraffin-embedded (FFPE) tumor blocks for pathological observation and gene detection. Diagnosis of patients with gastric tumor was confirmed by histopathology. Clinical and histopathological data, including age, sex, histology, tumor site, stage, histological grade and lymph node metastasis were all collected. Clinical characteristics of the patients were summarized in Table 1. Informed consent was obtained from all patients and the protocols for this study were approved by the Human Research Protective Committee of Drum Tower Hospital Affiliated to Medical School of Nanjing University. All animal experiments were performed in accordance with the Chinese Coordinating Committee on Cancer Research Regulations for the Welfare of Animals and the Animal Protection Law.

**Table 1:** Patient characteristics

**Characteristic**	**Training set (N = 100)**	**Independent testing set (N = 75)**	**In total (N = 175)**
Age, y median (range)	63 (29–83)	63 (29–83)	63 (29–83)
≥ 63	51 (51%)	40 (53%)	91 (52%)
< 63	49 (49%)	35 (47%)	84 (48%)
Sex			
Male	74 (74%)	57 (76%)	131 (75%)
Female	26 (26%)	18 (24%)	44 (25%)
Tumor Site			
Distal stomach	34 (34%)	28 (37%)	62 (35%)
Proximal stomach	41 (41%)	29 (39%)	70 (40%)
Whole stomach	25 (25%)	18 (24%)	43 (25%)
Stage			
I	13 (13%)	7 (9%)	20 (11%)
II	20 (20%)	21 (28%)	41 (23%)
III	65 (65%)	45 (60%)	110 (63%)
IV	2 (2%)	2 (3%)	4 (3%)
Histological grade			
2	20 (20%)	16 (21%)	36 (21%)
3	47 (47%)	34 (46%)	81 (46%)
Mixed 1–2	3 (3%)	3 (4%)	6 (3%)
Mixed 2–3	30 (30%)	22 (29%)	52 (30%)
Lymph node metastasis			
No	21 (21%)	19 (25%)	40 (23%)
Yes	79 (79%)	56 (75%)	135 (77%)

### HDRA

HDRA procedures were performed as described previously [18]. Briefly, the fresh tumor tissues were washed and minced into small pieces to approximately 0.5 mm in diameter, which were then placed on prepared collagen (Health Design, Rochester, NY) surfaces in 24-well microplates. There were 8 parallel culture wells for irinotecan sensitivity testing and 8 parallel culture wells for control. After incubation for 7 days at 37°C (in a humidified atmosphere containing 95% air −5% CO_2_) in the presence of drugs dissolved with RPMI 1640 medium containing 20% fetal calf serum, 100 μl type I collagenase (0.1 mg/ml, Sigma) and MTT (5 mg/ml, Sigma) were added to each culture well and incubated for another 16 hours. Concentration of irinotecan was 20 μg/ml according to its peak plasma concentration (ppc) in patients [19]. After extraction with dimethyl sulfoxide (DMSO, Sigma), absorbance of the solution in each well was read at 540 nm. Absorbance per gram of cultured tumor tissue was calculated from the mean absorbance of tissue from 8 parallel culture wells, and the tumor-tissue weight was determined before culture. The inhibition rate was calculated by using the following formula:

$$\mathrm{Inhibition}\;\mathrm{rate}\;\left(\%\right)=\left(1-T/C\right)\times 100\%$$

T is the mean absorbance of treated tumor/Weight

C is the mean absorbance of control tumor/Weight

### mRNA expression level detection

#### Total RNA extraction from FFPE tissue

Six 7-μm sections were prepared from FFPE tumor blocks that contained at least 80% tumor cells. After hematoxylin-eosin staining, the cancerous parts were microdissected and transferred into a microcentrifuge tube. RNA was isolated in accordance with a proprietary procedure (European patent number EP1945764-B1). Briefly, paraffin was removed by xylene, and microdissected cancerous parts were lysed in a proteinase K-containing buffer at 60°C for 16 h. RNA was purified by phenol and chloroform extractions followed by precipitation with isopropanol in the presence of sodium acetate at −20°C. The RNA pellet was washed in 70% ethanol and resuspended in 53 μl of RNase-free water followed by treatment with DNase I (Life Technologies).

#### QPCR assessment of gene expression

M-MLV Reverse Transcriptase Kit (Invitrogen) was applied to generate cDNA for Quantitative polymerase chain reaction (qPCR) to detect the β-actin (ACTB), APTX, BRCA1, ERCC1, ISG15 and Topo1. Each batch of reaction included a positive control from commercial human lung and liver RNA (Stratagene, La Jolla, CA, USA) as calibrators and negative controls without RNA and reverse transcriptase. Total RNA 1 μg was used for each RT reaction. Template cDNA was amplified with specific primers and probes for ACTB, APTX, BRCA1, ERCC1, ISG15 and Topo1 using Taqman Universal Master Mix (Applied Biosystems, Foster City, CA). The Assay IDs (Applied Biosystems, Foster City, CA) for the primers and probes were as follows: Hs99999903_m1 (ACTB), Hs00214452_m1 (APTX), Hs00157415_m1 (ERCC1), Hs00192713_m1 (ISG15), Hs00243257_m1 (Topo1), BRCA1 (NM_007294): forward 5’ GGCTATCCTCTCAGAGTGACATTTTA 3’, reverse 5’ GCTTTATCAGGTTATGTTGCATGGT 3’, and probe 6FAM −5’ CCACTCAGCAGAGGG 3’ MGB. QPCR was performed to quantify gene expression using the ABI Prism 7900HT Sequence Detection System (Applied Biosystems). The PCR conditions were 50°C for 2 min, 95°C for 15 min, followed by 40 cycles at 95°C for 15 s and 60°C for 1 min. Relative gene expression quantifications were calculated according to the comparative Ct method using ACTB as an endogenous control, based on our previous experience comparing different housekeeping genes [7,20], and commercial human lung and liver RNAs (Stratagene, La Jolla, CA, USA) as calibrators, which enables us to compare gene expression levels between different patients. Final results were determined by the formula mRNA expression level = 2^-(dCt sample-dCt calibrator) ^(dCt = Ct_gene_- Ct_ACTB_) [7,21] and were analyzed with the Stratagene analysis software.

#### DNA methylation detection

##### DNA extraction and modification

Three 7-μm sections were prepared from primary tumor blocks that contained at least 80% tumor cells. After hematoxylin-eosin staining, the cancerous parts were microdissected and transferred into a microcentrifuge tube. DNA was isolated routinely and then was chemically modified by sodium bisulphite to convert all unmethylated cytosines to uracils while leaving methylcytosines unaltered [16]. Then they were stored at −20°C for further analysis.

#### Methylation-specific polymerase chain reaction (MSP)

MSP was performed to determine the methylation of SULF2 using the ABI Prism 7300HT Sequence Detection System (Applied Biosystems). Each PCR reaction contained genomic DNA 2 μl, SYBR Green PCR Mix (TaKaRa, Japan) 10 μl, water 7.7 μl, and primers 0.15 μl (10 μmol/ l). The PCR conditions were 95°C for 10 min, followed by 45 cycles at 59°C for 30 s, 72°C for 30 s and 95°C for 30 s. Primers for SULF2 methylated PCR (TaKaRa, Japan) were as follows: forward 5’ TAAGTGTTTTTTTTATAGCGGC 3’, reverse 5’TACCGTAATTTCCGCTATC 3’. Primers for SULF2 unmethylated PCR (TaKaRa, Japan) were as follows: forward 5’ GTTTATAAGTGTTTTTTTATAGTGGT3’, reverse 5’TACCATAATTTCCACTATCCCT 3’. Each batch of reaction included a positive control from Methyltransferase (M.SssI)-treated human genomic DNA (fully methylated), a negative control from DNA samples which has been confirmed unmethylated and another negative control without DNA. All tests were performed in duplicate.

#### The establishment and validation of the gene-expression model for irinotecan sensitivity prediction

We adopted multiple linear regression analysis to establish the optimized gene-expression model based on the training set of 100 gastric cancers [22]. According to the results of stepwise regression (entry: α = 0.10, remove: α = 0.15), model consisted of APTX, Topo1 and BRCA1 is the optimized one. We assigned each patient an index according to the linear combination of the expression level of the mRNA weighted by the regression coefficient from the training samples. The index of the gene-expression model was calculated as follows: Index =0.488 - 0.020× expression level of APTX + 0.015× expression level of Topo1 - 0.011 × expression level of BRCA1. This model was later validated in another independent testing set of 75 patients with gastric cancer. In the testing set, patients were ranked according to their gene signature index and divided into sensitive-signature and resistant-signature groups by using the median index as the cutoff point. The irinotecan sensitivity of these two groups were tested by HDRA and compared with each other.

#### *In viv*o validation of the gene-expression model for irinotecan sensitivity prediction

To establish immunodeficient mice models with patient-derived gastric cancer xenografts, each freshly-removed surgical tumor tissue was cut into pieces of 3 × 3 × 3 mm^3^, which were transplanted within 30 min to 12 athymic immunodeficient mice, termed a “cohort” [23]. In each cohort, when the tumor grew to a size of 50–100 mm^3^, mice with xenografts were randomized to treatment with irinotecan 20 mg/kg/w, ip (n = 6) or no treatment as the control (n = 6). Individual tumor volumes (V) were calculated by the formula “V = (length × width × width)/2” and compared to the values at the start of treatment to obtain the relative tumor volume. Mice were observed every other day for tumor growth.

In order to evaluate the consistent inhibition of irinotecan in the sensitive-signature mice, three weeks after first administration, all the tumors were separated from the first generation mice and passaged to second generation mice. In the second generation, no drug was administrated. Mice were observed every day for another two weeks.

#### Statistical analysis

The Mann–Whitney U-test and the Kruskal-Wallis test were used to test the association between mRNA expression levels and clinical characteristics, and the association between irinotecan sensitivity and patients’ clinicopathological parameters. The Spearman’s rank method was used to assess the correlation of the mRNA expression levels between different genes as well as the correlation between mRNA levels and *in vitro* irinotecan sensitivity. The Mann–Whitney U-test was used to compare irinotecan sensitivity between SULF2M and SULF2U groups, irinotecan-sensitive and irinotecan-resistant patients and between sensitive-signature and resistant-signature groups. Receiver operating characteristic (ROC) curves were generated to calculate the sensitivity and specificity of prediction based on different genes and the gene-expression model in terms of irinotecan sensitivity. Paired Student’s t test was used to evaluate the differences between the tumor sizes of sensitive-signature mice or resistant-signature mice and controls. A *P* < 0.05 was considered statistically significant (two-sided). Statistical analysis was performed using the SPSS, version 16.0.

## Results

### Patient characteristics

Characteristics of all patients are shown in Table 1. In the 175 patients, the majority of patients were males (75%), and the histology of every sample was adenocarcinoma. In 62 (35%) patients, the tumor was located in the distal stomach, in 70 (40%) in the proximal stomach, and in 43 (25%) in the whole stomach. One hundred and ten (63%) patients had stage III disease. Lymph node metastasis was present in 135 (77%) patients.

### Gene expression levels

**Figure 1:**
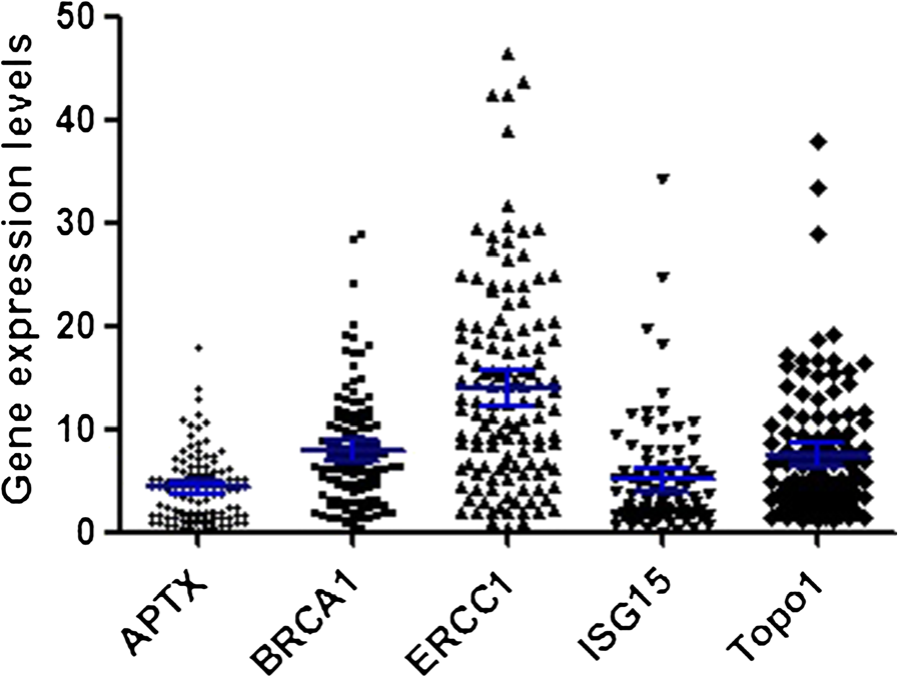
**Gene expression levels of APTX, BRCA1, ERCC1, ISG15 and Topo1 in 175 patients analyzed by quantitative RT-PCR.** Values of gene expression were calculated according to the comparative Ct method using ACTB as an endogenous control. The blue line stood for mean with 95% CI.

The mRNA expression levels of APTX, BRCA1, ERCC1, ISG15 and Topo1 were detected in all tumors, with median gene expression level relative to housekeeping ACTB of 4.32 for APTX (range 0.26–17.99, 95% confidence interval (CI): 3.78–5.19), 7.91 for BRCA1 (range 0.37–29.04, 95% CI: 7.07–9.51), 14.03 for ERCC1 (range 0.33–46.30, 95% CI: 13.01–17.07), 5.07 for ISG15 (range 0.04–34.24, 95% CI: 3.94–6.21), and 7.51 for Topo1 (range 1.07–37.81, 95% CI: 6.38–9.17) (Figure 1). A significant association was observed between APTX mRNA expression levels and histological grade (*P* = 0.03), and ISG15 mRNA and gender (*P* = 0.017). No other association between clinical characteristics and tumor mRNA levels was found (Table 2). However, a strong correlation was observed between the mRNA expression levels of APTX and BRCA1 (rho = 0.53, *P* < 0.001), APTX and ERCC1 (rho = 0.73, *P* < 0.001), BRCA1 and ERCC1 (rho = 0.48, *P* < 0.001) in tumor.

**Table 2:** The association of the gene expressions and pathological characteristics

**Characteristic**	**No. of Patients**	**APTX mRNA**	**BRCA1 mRNA**	**ERCC1 mRNA**	**ISG15 mRNA**	**Topo1 mRNA**
		**mean ± SD**	**mean ± SD**	**mean ± SD**	**mean ± SD**	**mean ± SD**
Age, y						
≥ 63	91 (52%)	4.81 ± 3.78	8.61 ± 5.73	15.28 ± 10.01	4.48 ± 3.69	8.03 ± 7.30
< 63	84 (48%)	4.09 ± 3.76	7.90 ± 5.95	14.75 ± 9.28	5.79 ± 6.90	7.47 ± 5.81
Sex						
Male	131 (75%)	4.77 ± 3.33	8.41 ± 5.57	15.74 ± 10.19	5.54 ± 5.62	7.95 ± 7.17
Female	44 (25%)	3.58 ± 3.29	7.92 ± 6.67	12.77 ± 7.30	3.53 ± 4.27*	7.21 ± 4.62
Tumor Site						
Distal stomach	62 (35%)	4.79 ± 2.87	8.97 ± 6.52	16.12 ± 10.36	7.05 ± 7.75	8.09 ± 5.93
Proximal stomach	70 (40%)	4.16 ± 3.10	8.33 ± 5.72	13.97 ± 9.09	3.61 ± 2.64	7.40 ± 6.63
Whole stomach	43 (25%)	4.60 ± 4.44	7.12 ± 4.74	15.31 ± 9.70	4.61 ± 3.43	7.06 ± 7.94
Stage						
I	20 (11%)	4.25 ± 4.47	5.91 ± 3.40	13.40 ± 7.07	1.89 ± 0.83	5.89 ± 4.03
II	41 (23%)	4.19 ± 3.01	8.58 ± 5.09	14.70 ± 9.56	6.76 ± 7.96	8.91 ± 6.35
III	110 (63%)	4.66 ± 3.38	8.19 ± 5.75	15.56 ± 10.16	4.67 ± 4.01	7.70 ± 7.07
IV	4 (3%)	4.20 ± 4.61	15.88 ± 17.90	11.01 ± 5.00	7.25 ± 6.33	4.03 ± 1.63
Histological grade						
2	36 (21%)	5.05 ± 3.54*	10.97 ± 7.33	15.78 ± 9.52	4.86 ± 4.75	8.08 ± 6.61
3	81 (46%)	3.92 ± 3.55	7.81 ± 5.16	14.30 ± 9.64	3.68 ± 2.75	7.18 ± 6.08
Mixed 1–2	6 (3%)	3.63 ± 4.51	7.17 ± 2.13	15.17 ± 12.40	8.00 ± 8.94	8.42 ± 6.02
Mixed 2–3	52 (30%)	4.55 ± 2.53	7.06 ± 5.27	15.65 ± 9.96	7.16 ± 7.74	8.44 ± 7.18
Lymph node metastasis						
No	40 (23%)	4.58 ± 3.39	8.70 ± 5.08	14.68 ± 7.31	5.97 ± 8.17	7.48 ± 4.47
Yes	135 (77%)	4.46 ± 3.35	8.16 ± 6.05	15.16 ± 10.33	4.78 ± 4.12	7.87 ± 7.23

* *P* < 0.05.

### The relationship between gene expression and chemosensitivity to irinotecan

**Figure 2:**
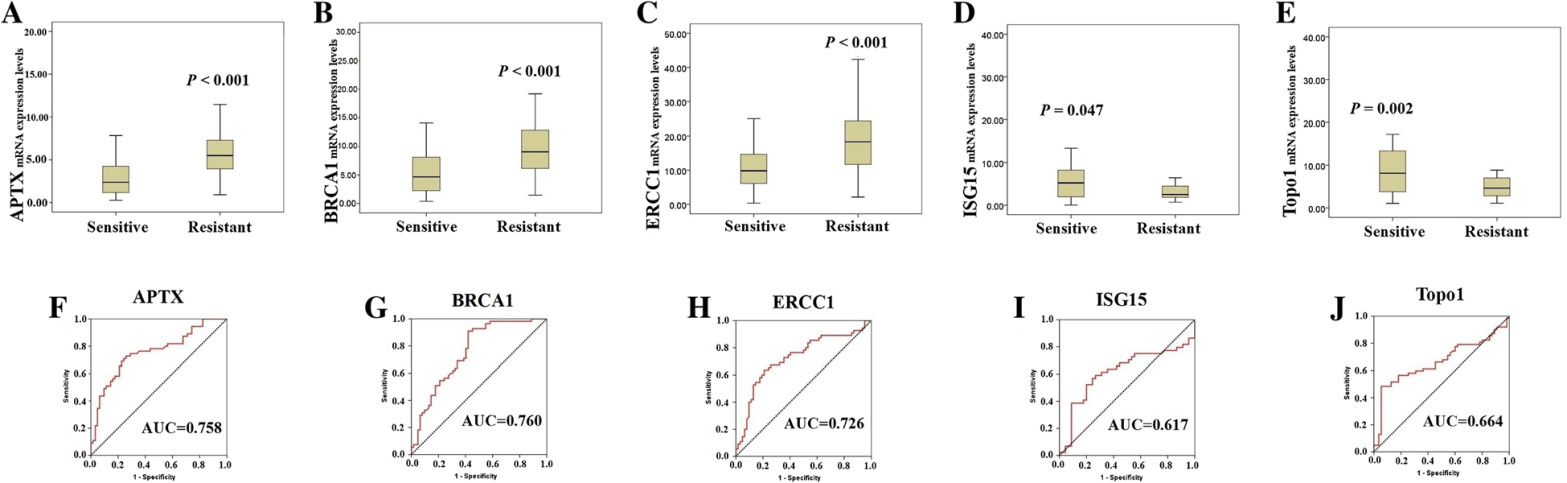
**Gene expression levels of APTX (*P*** **< 0.001), BRCA1 (*P*** **< 0.001) and ERCC1 (*P*** **< 0.001) were significantly lower in irinotecan-sensitive patients than in irinotecan-resistant patients, while ISG15 (*P*** **= 0.047) and Topo1 (*P*** **= 0.002) were significantly higher.** Box plots showed the mRNA expression levels of APTX (**A**), BRCA1 (**B**), ERCC1 (**C**), ISG15 (**D**) and Topo1 (**E**) in irinotecan-sensitive and irinotecan-resistant groups, respectively (n = 100). The lines inside the boxes denoted the medians. The whiskers of box plots: Min to Max. Graphs of ROC curve showed the AUCs of APTX (**F**), BRCA1 (**G**), ERCC1 (**H**), ISG15 (**I**) and Topo1 (**J**) for predicting irinotecan sensitivity. Sensitivity (Y-axis) was plotted against false-positive fraction (1 - specificity).

In the training set, the irinotecan sensitivity was successfully tested in all tumors, with median inhibition rate of 43.3% (range 2%–89%, CI: 39%–47%). There was no significant association between irinotecan sensitivity and clinical characteristics (Table 3), including age (*P =* 0.51), sex (*P =* 0.77), tumor site (*P =* 0.64), stage (*P =* 0.41), histological grade (*P =* 0.48) and lymph node metastasis (*P =* 0.47). However, mRNA levels of APTX (rho = −0.48, *P* < 0.001), BRCA1 (rho = −0.49, *P* < 0.001), ERCC1 (rho = −0.42, *P* < 0.001), ISG15 (rho = 0.34, *P* = 0.001), and Topo1 (rho = 0.43, *P* < 0.001) showed a correlation to irinotecan sensitivity. Patients were ranked according to their irinotecan inhibition rates and divided into irinotecan-sensitive and irinotecan-resistant groups by using the median inhibition rate as the cutoff point [19]. Gene expression levels of APTX (*P* < 0.001), BRCA1 (*P* < 0.001) and ERCC1 (*P* < 0.001) were significantly lower in irinotecan-sensitive patients than in irinotecan-resistant patients, while ISG15 (*P* = 0.047) and Topo1 (*P* = 0.002) were significantly higher (Figure 2A-E). ROC curves were generated to calculate the sensitivity and specificity of the each gene in predicting irinotecan sensitivity (Figure 2G-J). The areas under the ROC curve (AUC), sensitivity and specificity for the prediction of irinotecan sensitivity based on APTX, BRCA1, ERCC1, ISG15 and Topo1 mRNA levels were listed in Table 4.

**Table 3:** Association between irinotecan sensitivity and clinical characteristics

**Characteristic**	**Irinotecan inhibition rate**	**Irinotecan inhibition rate**
	**mean (95% CI)**	**mean (95% CI)**
	**Training set (N = 100)**	**Independent testing set (N = 75)**
Age, y median (range)		
≥ 63	42% (36–47%)	44% (31–57%)
< 63	45% (39–51%)	44% (34–54%)
Sex		
Male	43% (38–47%)	42% (33–51%)
Female	45% (36–55%)	50% (31–68%)
Tumor Site		
Distal stomach	42% (35–49%)	44% (28–60%)
Proximal stomach	44% (38–51%)	47% (35–58%)
Whole stomach	43% (35–51%)	37% (16–59%)
Stage		
I	34% (21–47%)	36% (3–70%)
II	49% (40–57%)	50% (31–68%)
III	43% (38–48%)	44% (34–54%)
IV	33%	34%
Histological grade		
2	41% (33–49%)	44% (21–68%)
3	42% (36–48%)	39% (27–51%)
Mixed 1–2	54%	58%
Mixed 2–3	46% (38–54%)	49% (35–62%)
Lymph node metastasis		
No	42% (33–51%)	46% (27–65%)
Yes	44% (39–48%)	44% (34–53%)
Signature index		
> 0.43	57% (52–63%) **	65% (61–70%) **
≤ 0.43	31% (27–36%)	22% (17–28%)

** *P* < 0.001.

**Table 4:** The sensitivity and specificity of five gene expression levels for the prediction of irinotecan sensitivity

**Genes**	**Chemotheraputic agents**	**Sensitivity**	**Specificity**	**AUC (95% CI)**	***P***
APTX	Irinotecan	73%	74%	0.758 (0.669-0.847)	<0.001
BRCA1	Irinotecan	91%	58%	0.760 (0.673-0.846)	< 0.001
ERCC1	Irinotecan	64%	79%	0.726 (0.632-0.821)	<0.001
ISG15	Irinotecan	59%	73%	0.617 (0.494-0.740)	0.047
Topo1	Irinotecan	48%	94%	0.664 (0.563-0.765)	0.002

### The relationship between SULF2 methylation and sensitivity to irinotecan

In the training set, the methylation status of SULF2 was successfully detected in all patients, with thirty-three (28%) carrying SULF2M and eighty-four (72%) carrying SULF2U. There was no significant association between SULF2 methylation status and clinical characteristics. The irinotecan inhibition rates were 49.8% (range 2%–89%, 95% CI: 41%–59%) for SULF2M group, and 40.2% for SULF2U group (range 2%–84%, 95% CI: 34%–46%, *P* = 0.08).

### The establishment of the gene-expression model and its association with sensitivity to irinotecan

**Figure 3:**
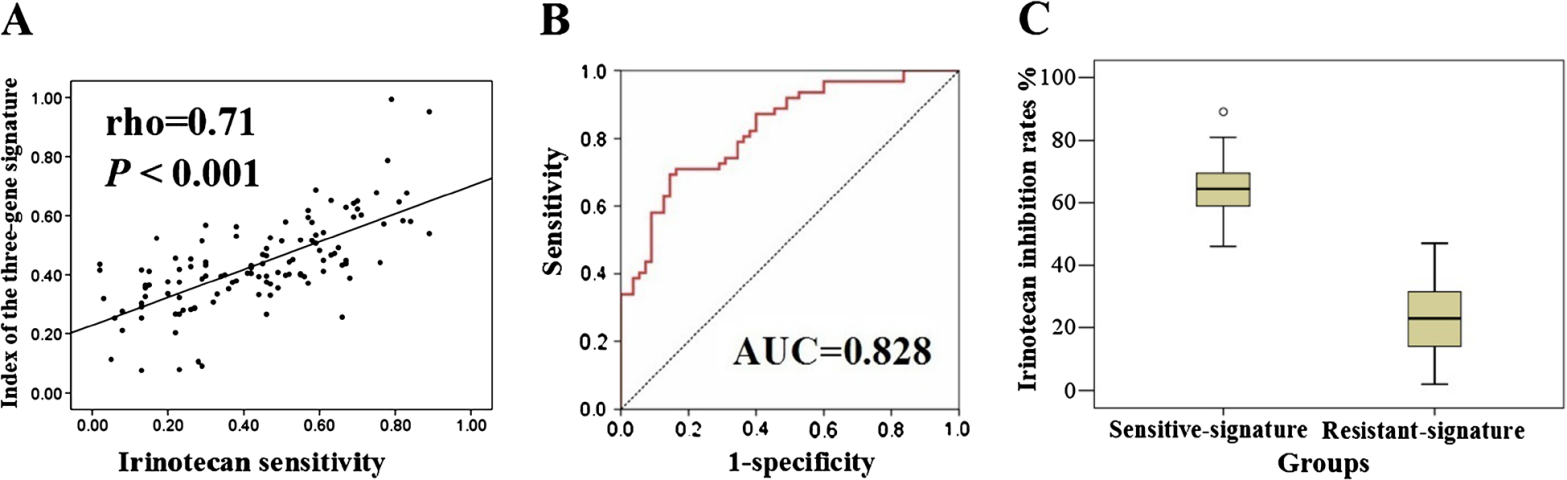
**The evaluation and validation of the three-gene signature for irinotecan sensitivity prediction. A**, there was a significant association between the three-gene signature index and irinotecan sensitivity (rho = 0.71, *P* < 0.001). Index (Y-axis) was plotted against irinotecan inhibition rate. “0.00” stood for no inhibition, while “1.00” stood for 100% inhibition. **B**, ROC curve was made for irinotecan sensitivity prediction based on the three-gene signature index. Sensitivity (Y-axis) was plotted against false-positive fraction (1 - specificity). The AUC was 0.828 (95% CI: 0.755–0.901, *P* < 0.001). **C**, the irinotecan inhibition of gastric samples with sensitive-signature was much higher than those with resistant-signature (65% vs. 22%, *P* < 0.001). Sensitive-signature group: three-gene signature index > 0.43, n = 37; resistant-signature group: index ≤ 0.43, n = 38.

Based on the expression level of five genes and status of SULF2 methylation, we constructed a signature by multiple linear regression analysis as mentioned in the methods. The index of the three-gene signature ranged from 0.08 to 0.99 with mean value of 0.43 ± 0.16. There was a significant correlation between the index and irinotecan sensitivity (rho = 0.71, *P* < 0.001) (Figure 3A). ROC curve was generated to calculate the sensitivity and specificity of the three-gene signature in predicting irinotecan sensitivity (Figure 3B). The AUC was 0.828 (95% CI: 0.755–0.901, *P* < 0.001). With the threshold value of 0.43, the sensitivity and specificity for the prediction of irinotecan sensitivity based on the three-gene signature reached 73% and 86%, respectively.

### Validation of the three-gene signature in the independent testing set

To determine if the three-gene signature could significantly distinguish the sensitive and resistant group to irinotecan, we applied it to an independent testing set including 75 gastric adenocarcinomas. All the patients’ characteristics were listed in Table 1. In the testing set, the irinotecan sensitivity was successfully tested in all tumors, with median inhibition rate of 44.0% (range 2%–89%, CI: 37%–51%). There was no significant association between irinotecan sensitivity and clinical characteristics (Table 3), including age (*P =* 0.54), sex (*P =* 0.81), tumor site (*P =* 0.54), stage (*P =* 0.61), histological grade (*P =* 0.52) and lymph node metastasis (*P =* 0.79). The mRNA expression levels of APTX, BRCA1, ERCC1, ISG15 and Topo1 were detected in all tumors and the gene signature index was calculated as mentioned above. We divided those patients into two groups according to their gene signature index (sensitive-signature group: index > 0.43, n = 37; resistant-signature group: index ≤ 0.43, n = 38). The irinotecan inhibition rates in gastric samples with sensitive-signature were much higher than those with resistant-signature (65% vs. 22%, *P* < 0.001) (Table 3, Figure 3C). There was a significant correlation between the index and irinotecan sensitivity (rho = 0.79, *P* < 0.001). ROC curve was generated to calculate the sensitivity and specificity of the three-gene signature in predicting irinotecan sensitivity in this set of patients. The AUC was 0.939 (95% CI: 0.882–0.996, *P* < 0.001). With the threshold value of 0.43, the sensitivity and specificity for the prediction of irinotecan sensitivity based on the three-gene signature reached 84% and 94%, respectively.

### *In vivo* validation of the three-gene signature

**Figure 4:**
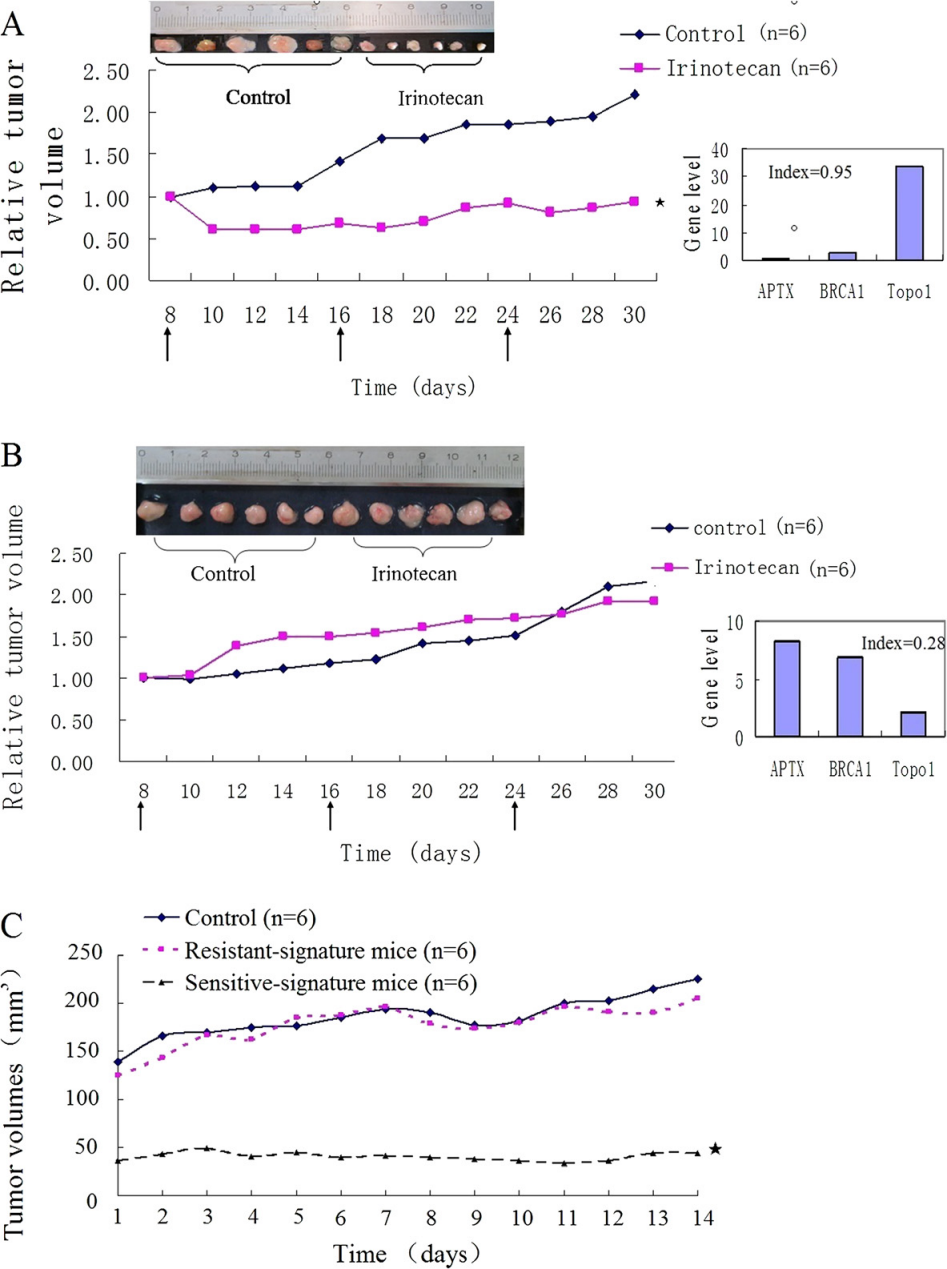
**Response to irinotecan in patient-derived gastric cancer xenografts with different three-gene signature. A**, chemosensitivity of mice model with sensitive-signature (Index = 0.95, gene expression level: APTX = 1.01, BRCA1 = 2.78 and Topo1 = 33.96). **B**, chemosensitivity of mice model with resistant-signature (Index = 0.28, gene expression level: APTX = 8.33, BRCA1 = 6.87 and Topo1 = 2.09). Twelve mice per cohort were treated with the irinotecan or no administration in control as described in Materials and Methods. The black arrow stood for administration time. Tumor growth was measured every other day, and relative tumor volume (Y-axis) was plotted against time. **C**, in the second generation, the subsequent tumor size in sensitive-signature mice kept exhibiting a reduced tumor size compared with the tumor size in resistant-signature and control groups. Tumor growth was measured every day, and tumor volumes (Y-axis) were plotted against time. SDs were not shown because of better clarity. Asterisks (★), significance compared with control.

Twenty cohorts of immunodeficient mice (12 mice per cohort, 240 mice in total) with human-derived xenografts were successfully established from the 75 surgical specimens of the independent testing set. Based on the mRNA expression of APTX, BRCA1 and Topo1, one cohort of mice (n = 12) carrying surgical tumors with sensitive-signature (Index = 0.95, gene expression level: APTX = 1.01, BRCA1 = 2.78 and Topo1 = 33.96) and another cohort (n = 12) with resistant-signature (Index = 0.28, gene expression level: APTX = 8.33, BRCA1 = 6.87 and Topo1 = 2.09) were chosen for *in vivo* validation of the three-gene signature. There were 12 mice in each cohort (six for irinotecan administration and six for control). Irinotecan therapy with 20 mg/kg per week to immunodeficient mice carrying xenografts with sensitive-signature was well tolerated and dramatically arrested the growth of tumors (*P* < 0.001, Figure 4A), but there was no effect for the same treatment on mice carrying xenografts with resistant-signature (*P* = 0.83, Figure 4B).

Three weeks after first irinotecan administration, all the tumors were separated from the first generation mice and passaged to the second generation. There were in total three groups in the second generation: mice carrying xenografts with sensitive-signature and having been treated with irinotecan (named “sensitive-signature mice” for short, n = 6), mice carrying xenografts with resistant-signature and having been treated with irinotecan (named “resistant-signature mice” for short, n = 6) and the controls (no irinotecan administration before, n = 6). The subsequent tumor size in sensitive-signature mice kept exhibiting a reduced tumor size compared with the tumor size in resistant-signature and control groups (sensitive-signature mice vs. control: *P* < 0.001; resistant-signature mice vs. control: *P* = 0.10, Figure 4C).

## Discussion

The screening and validation of molecular biomarkers capable of predicting response to different chemotherapeutic agents constitutes a significant step towards personalized treatment for cancer patients. In the field of prognostic biomarkers, advances in genome-wide sequencing and microarray analysis have allowed the identification of molecular signatures that can promote more precise classification and prognostication of human cancers [24-26]. It was reported that a five-gene signature was closely associated with relapse-free and overall survival among patients with NSCLC, and a 54-gene signature could predict the risk of recurrence in NSCLC [25]. A 21-gene signature also has been demonstrated to predict the risk of distant recurrence in postmenopausal patients with breast cancer treated with anastrozole or tamoxifen [26]. However, up to now, in the field of chemosensitivity predictive biomarkers, there is still no such gene signature for personalizing chemotherapy in gastric cancer.

In the present study, we firstly investigated the value of APTX, BRCA1, ERCC1, ISG15, SULF2 and Topo1 as predictive biomarkers to irinotecan, respectively. We found that although those genes had correlation with irinotecan sensitivity, their function in irinotecan sensitivity prediction was limited and their combination might improve efficiency. Therefore, we established a three-gene signature by multiple linear regression analysis and demonstrated the promising value of this signature in distinguishing two subgroups of advanced gastric cancer patients that widely differed in their sensitivity to irinotecan treatment. Moreover, validation was carried out in another independent testing set and two cohorts of immunodeficient mice models with patient-derived gastric cancer xenografts. It was showed that samples with sensitive-signature were significantly more sensitive to irinotecan than those with resistant-signature. Immunodeficient mice model with human-derived xenograft also showed that mice with sensitive-signature (high three-gene signature index) could benefit from irinotecan therapy dramatically and were delayed in tumor growth in the second generation; whereas mice with resistant-signature (low three-gene signature index) had no response to irinotecan and their tumor kept growing in both first and second generation. Taken together, these findings indicate that this three-gene signature is closely associated with irinotecan sensitivity among patients with gastric cancer.

The chemosensitivity assay we adopted in the present study included HDRA for *in vitro* testing and immunodeficient mice models with patient-derived gastric cancer xenografts for *in vivo* validation. HDRA has been demonstrated by varieties of studies as a useful predictor for chemosensitivity at different cancerous sites, including gastrointestinal cancer [18]. It has been reported in gastric cancer[18], esophageal cancer [19], breast cancer [27], oral squamous cell carcinomas [28] and head and neck cancer [29] that efficacy rate for an individual agent using HDRA assay *in vitro* has a considerable good correlation with clinical response rate to each agent. The value of patient-derived tumor xenograft model has been investigated and evaluated in various studies, including retrospective and prospective clinical studies [23,30-33]. Similar to the original tumor sample in histological and gene status, the response of xenograft models could predict the efficiency of chemotherapeutic agents in more than 90% patients [23,30]. Good correlations between efficacy rate for an individual agent using such model and clinical response rate to each agent have been well demonstrated [31]. A patient with advanced and gemcitabine-resistant pancreatic cancer resulted in long-lasting tumor response after the efficient treatment guided by the personalized xenograft model generated from the patient’s freshly-removed tumor [32]. In another pilot clinical study, patients with advanced cancer were treated with 17 selected regimens on the basis of personalized tumor grafts. Consequently, durable partial remissions were observed in 15 cases [33]. These results supported the notion of patient-derived tumor xenograft models as a powerful platform for chemosensitivity evaluation. In present study, we established different cohorts of immunodeficient mice models with patient-derived gastric cancer xenografts, and demonstrated that tumor growth were significantly suppressed in the cohort with sensitive-signature (low APTX and BRCA1, but high Topo1 mRNA expression level, Index = 0.95) when treated with irinotecan, but had no differences compared with cohort with resistant-signature (high APTX and BRCA1, but low Topo1 mRNA expression level, Index = 0.28). The results of the second generation tumor showed that irinotecan might have anti-cancer efficiency on stem-like cells and therefore the tumor growth was delayed in the second generation of the sensitive-signature group.

We have to admit that HDRA and mice model might still not be representative of the behavior of the patient’s tumors because of the cancer heterozygote and patients’ characteristics, such as age, gender, tumor size and location. There may unavoidably be an imperfect relationship between tumor response and survival because of treatment associated adverse events. In order to avoid tumor heterogeneity, we designed 8 parallel culture wells for irinotecan sensitivity testing and 8 parallel culture wells for control from different parts of one patient’s tumor sample. The mice models we have established were derived from 75 patients with different clinicopathological parameters. We will follow up those patients to confirm that whether this *in vivo* and *in vitro* inhibition would have consequences for the therapy as well as patients’ outcome.

Gene-expression signature usually established by the use of microarrays and later validated by qPCR [24]. However, in clinical practice, microarrays usually involving a large number of genes in the analysis are limited by complicated methods, lack of reproducibility, the need for fresh-frozen tissues and further independent validation of the results [34]. RT-PCR comprising a smaller number of genes may be more clinically useful, allowing for reproducible and accurate quantification of results for small amounts of RNA obtained from FFPE specimens [24,34]. In the current study, we firstly selected six candidate predictive biomarkers for irinotecan based on widely literature review and previous investigation, and then RT-PCR were performed for gene detection and further analysis. The method we adopted to detect mRNA expression levels in FFPE specimens is feasible for routine gene expression analysis in daily clinical practice.

According to the results of stepwise regression, the predictive model we established consists of three genes (APTX, BRCA1 and Topo1) finally. mRNA levels of APTX and BRCA1 are both negatively correlated with irinotecan sensitivity, while Topo1 level is positively correlated with irinotecan sensitivity. Irinotecan, as a kind of Topo1 inhibitors, can stabilize of Topo1-DNA complex that upon collision with the replication fork causes double-strand DNA breaks, cell cycle arrest and death [12]. Therefore, the direct molecular target Topo1 was regarded as the best-characterized biomarker capable of predicting response to irinotecan [14]. A clinical study in metastatic colorectal cancer has reported that higher protein levels of Topo1 were correlated longer overall survival (17.4 months vs. 14.7 months, *P* = 0.005) and better response to irinotecan significantly [14]. Staying with the same line of the previous study, the current study demonstrated that both singly or combined in the three-gene signature, tumors with higher mRNA levels of Topo1 were more sensitive to irinotecan in gastric cancer.

Irinotecan treatment results in the accumulation of DNA strand breaks in tumor cells, and APTX, BRCA1 and ERCC1 have been shown to have important roles in the repair of DNA single- and double-strand breaks [15]. Validation in a panel of 30 colorectal cancer cell lines, the levels of APTX were significantly associated with CPT sensitivity (*P* = 0.004) [35]. It also reported that APTX as a predictive biomarker was capable of identifying a subset of advanced colorectal cancer patients with high probability of response to irinotecan-based treatment. Patients with low levels of APTX had improved progression-free (9.2 vs. 5.5 months, *P* = 0.03) and overall survival (36.7 vs. 19 months, *P* = 0.008) [13]. Both BRCA1 and ERCC1 play central roles in nucleotide excision repair in DNA damage response pathways. BRCA1 has been identified as differential modulators of sensitivity to cisplatin and docetaxel [7]. BRCA1 was also been reported to be related with the sensitivity of Topo1 poison in a study of mice model with mammary tumors [36]. ERCC1 is part of the ERCC1–ERCC4 (XPF) heterodimeric structure-specific endonuclease, and has been implicated in platinum resistance. Recently, ERCC1 was also demonstrated by a cell line study to be involved in repair of CPT-induced DNA damage and had potential value in predicting CPT sensitivity [37]. As a supplement to the previous studies, the current study further demonstrated that higher APTX, BRCA1 and ERCC1 mRNA expression levels suggested lower likelihood of response to irinotecan-based chemotherapy in gastric cancer. The three-gene signature with APTX and BRCA1 could predict sensitivity to irinotecan more precisely. This may result from the reason that DNA damage caused by irinotecan would be repaired more efficiently when APTX, BRCA1 and ERCC1 expression in high levels, and therefore, these samples would have a poor response to this form of treatment. Moreover, the regression coefficient for APTX was higher than for Topo1, which might indicate that the response to irinotecan in gastric tumors could highly dependent on DNA repair mechanisms. The specific mechanisms remain to be further studied and elucidated.

SULF2 promotes growth and metastasis of solid tumors. It has been demonstrated that promoter CpG island methylation of SULF2 is highly prevalent in resected lung adenocarcinomas and is significantly associated with better survival [38]. ISG15 interferes with the ubiquitin/26S proteasome pathway and increase the sensitivity to Topo1 inhibitors by leading to accumulation of CPT-induced DNA damage and resulting in an increased level of apoptosis [17]. In NSCLC, silencing SULF2 through methylation could result the significant increase of ISG15 mRNA expression levels and increase sensitivity to Topo1 inhibitors *in vitro*[16]. In the present study, based on freshly-removed gastric tumors, ISG15 was demonstrated to correlate with irinotecan sensitivity positively. Samples with higher mRNA expression levels of ISG15 were more sensitive to irinotecan. Our study also showed that SULF2M group might have a higher likelihood of benefit from irinotecan-based treatment than SULF2U group. Further validation is warranted.

## Conclusion

In conclusion, the establishment of this three-gene signature as a new model predicting the sensitivity to irinotecan treatment constitutes a new step towards the goal of individualized treatment for gastric cancer patients. Our results suggest that a patient with a tumor that has high levels of the three-gene signature index would be an ideal candidate to receive single or combined treatment with irinotecan. These findings are preliminary and suggestive at this point, and this three-gene signature needs to be validated before being used in routine daily clinical practice. A clinical trial is currently being designed in order to validate the role of customizing treatment based on this three-gene signature.

## Abbreviations

FFPE: Formalin-fixed paraffin-embedded; HDRA: Histoculture drug response assay; APTX: Aprataxin; BRCA1: Breast cancer susceptibility gene 1; ERCC1: Excision repair cross-complementing 1; ISG15: INF-inducible regulator of ubiquitination; Topo1: Topoisomerase I; SULF2: Heparan sulfate 6-O-endosulfatase

## Competing interest

We declare that we have no conflicts of interest.

## Authors’ contributions

JS, JW, WXG and BRL designed the research and wrote the paper. JS, HW, GFY and LXY performed the research. YY, LX and ZZ analyzed data. JW, XPQ and YTD edited paper. All authors read and approved the final manuscript.
